# Global update on the susceptibility of human influenza viruses to neuraminidase inhibitors and status of novel antivirals, 2016–2017

**DOI:** 10.1016/j.antiviral.2018.07.001

**Published:** 2018-09

**Authors:** Angie Lackenby, Terry G. Besselaar, Rod S. Daniels, Alicia Fry, Vicki Gregory, Larisa V. Gubareva, Weijuan Huang, Aeron C. Hurt, Sook-Kwan Leang, Raphael T.C. Lee, Janice Lo, Lori Lollis, Sebastian Maurer-Stroh, Takato Odagiri, Dmitriy Pereyaslov, Emi Takashita, Dayan Wang, Wenqing Zhang, Adam Meijer

**Affiliations:** a National Infection Service, Public Health England, London, NW9 5HT, United Kingdom; b Global Influenza Programme, World Health Organization, Avenue Appia 20, 1211, Geneva 27, Switzerland; c The Francis Crick Institute, Worldwide Influenza Centre (WIC), WHO Collaborating Centre for Reference and Research on Influenza, 1 Midland Road, London, NW1 1AT, United Kingdom; d WHO Collaborating Center for Surveillance, Epidemiology and Control of Influenza, Centers for Diseases Control and Prevention (CDC), 1600 Clifton RD NE, MS-G16, Atlanta, GA, 30329, USA; e WHO Collaborating Centre for Reference and Research on Influenza, National Institute for Viral Disease Control and Prevention, China CDC, Beijing, China; f WHO Collaborating Centre for Reference and Research on Influenza, At the Peter Doherty Institute for Infection and Immunity, Melbourne, Victoria, 3000, Australia; g Department of Microbiology and Immunology, University of Melbourne, Parkville, Victoria, 3010, Australia; h Bioinformatics Institute, Agency for Science, Technology and Research, 30 Biopolis Street, #07-01, Matrix, Singapore, 138671, Singapore; i Public Health Laboratory Centre, Centre for Health Protection, Department of Health, 382 Nam Cheong Street, Hong Kong, China; j Department of Biological Sciences, National University of Singapore, 14 Science Drive 4, Singapore, 117543, Singapore; k National Public Health Laboratory, Ministry of Health, 3 Biopolis Drive, Synapse #05-14 to 16, Singapore, 138623, Singapore; l WHO Collaborating Centre for Reference and Research on Influenza, National Institute of Infectious Diseases, Gakuen 4-7-1, Musashimurayama, Tokyo, 208-0011, Japan; m Division of Communicable Diseases, Health Security, & Environment, World Health Organization Regional Office for Europe, UN City, Marmorvej 51, DK-2100, Copenhagen Ø, Denmark; n National Institute for Public Health and the Environment, PO Box 1, 3720 BA, Bilthoven, The Netherlands

**Keywords:** Influenza, Neuraminidase, Inhibitor, Susceptibility, Surveillance, Resistance

## Abstract

A total of 13672 viruses, collected by World Health Organization recognised National Influenza Centres between May 2016 and May 2017, were assessed for neuraminidase inhibitor susceptibility by four WHO Collaborating Centres for Reference and Research on Influenza and one WHO Collaborating Centre for the Surveillance Epidemiology and Control of Influenza. The 50% inhibitory concentration (IC_50_) was determined for oseltamivir and zanamivir for all viruses, and for peramivir and laninamivir in a subset (n = 8457). Of the viruses tested, 94% were obtained from the Western Pacific, Americas and European WHO regions, while limited viruses were available from the Eastern Mediterranean, African and South East Asian regions.

Reduced inhibition (RI) by one or more neuraminidase inhibitor was exhibited by 0.2% of viruses tested (n = 32). The frequency of viruses with RI has remained low since this global analysis began (2015/16: 0.8%, 2014/15: 0.5%; 2013/14: 1.9%; 2012/13: 0.6%) but 2016/17 has the lowest frequency observed to date. Analysis of 13581 neuraminidase sequences retrieved from public databases, of which 5243 sequences were from viruses not included in the phenotypic analyses, identified 58 further viruses (29 without phenotypic analyses) with amino acid substitutions associated with RI by at least one neuraminidase inhibitor. Bringing the total proportion to 0.5% (90/18915).

This 2016/17 analysis demonstrates that neuraminidase inhibitors remain suitable for treatment and prophylaxis of influenza virus infections, but continued monitoring is important. An expansion of surveillance testing is paramount since several novel influenza antivirals are in late stage clinical trials with some resistance already having been identified.

## Highlights

• A total of 13,672 influenza viruses were collected worldwide, May 2016–May 2017.
• Approximately 0.2% showed reduced inhibition by at least one NA inhibitor.
• The frequency of viruses with reduced inhibition by NA inhibitors was at the lowest since analysis began in 2012.
• Newly licenced antivirals and drugs currently in clinical trials are summarized.
• Global surveillance of influenza antiviral susceptibility should be continued.

## 1 Introduction

Neuraminidase inhibitors (NAI) remain the only widely licenced class of antiviral drugs appropriate for treatment and prophylaxis of seasonal influenza. Oseltamivir is extensively prescribed with zanamivir, peramivir and laninamivir being used in fewer countries consecutively (Ison, 2017). M2 inhibitors remain unsuitable for clinical use due to widespread resistance. Recent detection of M2 inhibitor-susceptible A(H3N2) viruses shows that continual surveillance is useful as there is potential for circulation of these susceptible viruses to expand (Bright et al., 2005; Hurt et al., 2017). The WHO Global Influenza Surveillance and Response System (GISRS) laboratories and GISRS experts in the WHO Antiviral Working Group (WHO-AVWG) perform surveillance of influenza antiviral susceptibility and enhance capability through the GISRS network, ensuring appropriate monitoring.

GISRS laboratories receive thousands of influenza virus-positive clinical specimens each year and isolate viruses for antigenic and antiviral susceptibility characterisation. This is the 5th WHO-AVWG annual review of NAI phenotypic susceptibility data generated by the five WHO Collaborating Centres (CCs) and neuraminidase (NA) sequence data from the CCs, National Influenza Centres (NICs) and other laboratories submitted to two public sequence repositories, the Global Initiative on Sharing All Influenza Data (GISAID; www.GISAID.org) and National Centre for Biotechnology Information Influenza Virus resource (NCBI-IVR; www.ncbi.nlm.nih.gov/genomes/FLU). Since the first global analysis of 2012/13 data, the proportion of reduced inhibition (RI) by NAI in seasonal influenza viruses has remained low, but variable across seasons (Gubareva et al., 2017a; Hurt et al., 2016; Meijer et al., 2014; Takashita et al., 2015). A(H1N1)pdm09 viruses with NA H275Y amino acid substitution (AAS) are the most frequently observed oseltamivir-resistant viruses.

The clinical significance of *in vitro* determined NAI susceptibility is unclear but correlation with IC_50_ value, the concentration of drug required to inhibit NA enzymatic activity by 50% is accepted (Zambon and Hayden, 2001). To this end, influenza type A viruses are considered as having RI by a NAI when exhibiting an increase in IC_50_ value, compared to a reference IC_50_ value (e.g. a median IC_50_ of viruses of the same type/subtype), of at least 10-fold and highly reduced inhibition (HRI) if increased 100-fold or greater (WHO, 2012). Thresholds are 5-fold for RI and 50-fold for HRI for influenza type B viruses due to higher baseline IC_50_ values. Gene sequencing is increasingly being adopted by laboratories as costs decrease. Analysis of all available NA sequences shows that there is a continual but sporadic emergence of viruses with AAS known to cause RI or HRI by one or more NAIs, with or without exposure to drug, and AAS affecting susceptibility is type and subtype specific. The WHO-AVWG has published a table of these AAS which is regularly reviewed (http://www.who.int/influenza/gisrs_laboratory/antiviral_susceptibility/avwg2014_nai_substitution_table.pdf).

A waxing and waning of AAS associated with RI/HRI by one or more NAIs has been observed over the years of global analysis. The phenotypic analysis performed by CCs, and some NICs, is critical for accurate interpretation of NA sequence data. The timely sharing of surveillance data on NAI susceptibility is essential to making informed decisions on patient management, and for strategic planning for pandemic preparedness by governments and public health bodies.

## 2 Overall analysis of phenotypic neuraminidase inhibitor susceptibility data from CCs

NICs receive and characterise influenza virus-positive clinical specimens in their respective countries. Representative numbers of virus isolates and/or clinical specimens by type, subtype and lineage are forwarded to at least one CC for further characterisation, according to the WHO terms of reference and referral guidance for NICs, available here: http://www.who.int/influenza/gisrs_laboratory/national_influenza_centres/tor_nic.pdf and http://www.who.int/influenza/gisrs_laboratory/seasonal_sharing_guide). The referral guidance criteria are actioned differently by NICs dependent on a variety of conditions including but not limited to the local influenza season severity and timing, the national testing capability and the level of use of NAIs in the country. Once received at the WHO CC, virus isolation and propagation is performed in MDCK and/or MDCK-SIAT1 cells prior to NAI susceptibility testing. The five WHOCCs perform phenotypic antiviral susceptibility analysis on all influenza viruses received or isolated. When possible, sequence analysis by Sanger or next generation sequencing of paired clinical specimen and virus isolate is performed when a RI/HRI phenotype is identified.

**Fig. 1:**
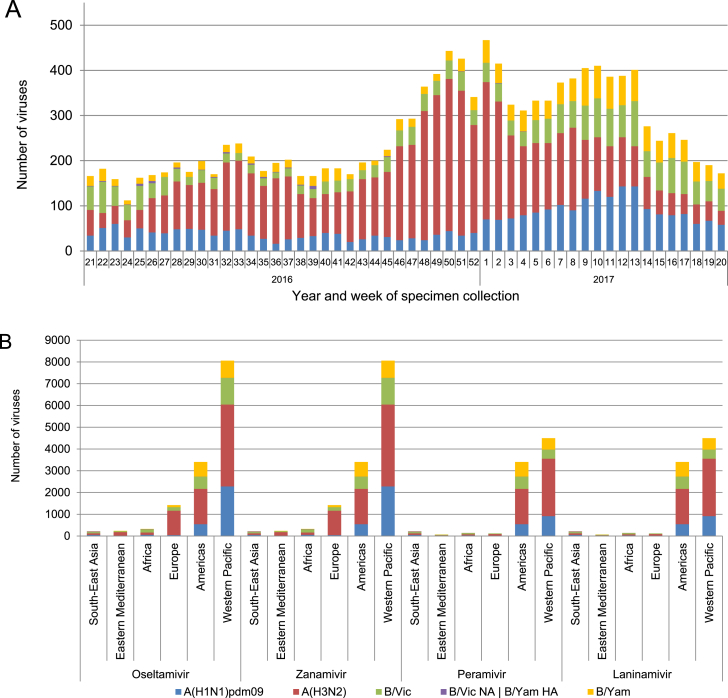
Influenza viruses collected and tested for phenotypic neuraminidase inhibitor (NAI) susceptibility during 2016–2017. A) Week of specimen collection and virus type/subtype/lineage; for specimens tested, peaks in specimen collection during the Southern Hemisphere winter and during the Northern Hemisphere winter were observed. B) Number of viruses tested for phenotypic susceptibility to the four NAIs by World Health Organization region. B/Yamagata-lineage haemagglutinin:B/Victoria-lineage neuraminidase reassortants are shown separately.

Viruses isolated from specimens collected between week 21/2016 (23 May 2016) and week 20/2017 (21 May 2017) are included in this analysis of phenotypic NAI susceptibility (Fig. 1A). A total of 13672 viruses were tested for susceptibility to oseltamivir and zanamivir, with a subset of these (n = 8457; 62%) also tested for susceptibility to peramivir and laninamivir (Fig. 1B). The majority of isolates tested originate from the WHO regions of Western Pacific (59%), American (25%) and European (10%) (Fig. 1B). Only 6% of viruses tested originate from the Eastern Mediterranean, African and South East Asian regions (2% per region). In total, 239482 influenza virus detections, globally, were reported to FluNet, the WHO GISRS global web-based tool for influenza virological surveillance (www.who.int/influenza/gisrs_laboratory/flunet/en) in the timeframe of this antiviral analysis. The viruses analysed for phenotypic susceptibility to NAIs by the WHO CCs in this study represent 2.8% of virus detections globally (2.5% of influenza A viruses, 3.9% of influenza B viruses). The proportion of viruses detected globally, that were tested for phenotypic susceptibility in this study varied by WHO region (SEARO: 2.9%; EMRO: 3.5%; AFRO: 6.1%; EURO: 0.9%; PAHO: 1.4%; WPRO: 9.5%).

**Fig. 2:**
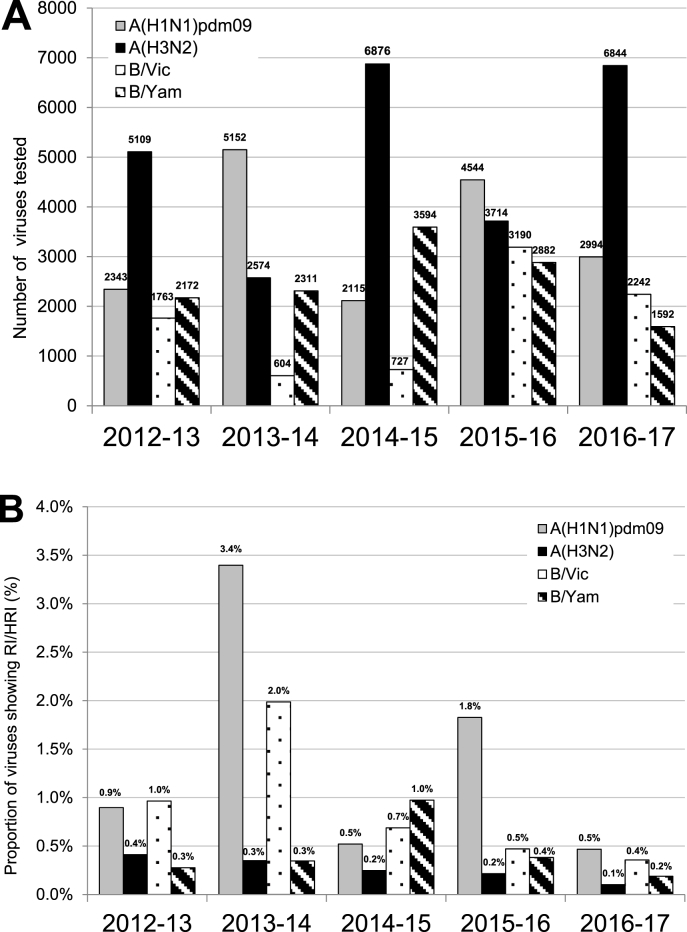
**Comparison of NAI susceptibility surveillance over five seasons**. A) Number of viruses tested in neuraminidase inhibition assays over the 2012–2017 period. B) Proportion of viruses showing RI or HRI by NAIs over the 2012–2017 period. Data compiled from the global studies reporting on viruses isolated during 2012–13 (Meijer et al., 2014), 2013–14 (Takashita et al., 2015), 2014–15 (Hurt et al., 2016), 2015–16 Gubareva et al., 2017a) and 2016–17 (current study). B/Yamagata-lineage haemagglutinin:B/Victoria-lineage neuraminidase reassortants are included in the proportion and number of B/Victoria-lineage viruses.

Influenza A(H3N2) viruses prevailed over this time period (6844; 50%) followed by A(H1N1)pdm09 (2994; 22%), B/Victoria-lineage (2242; 16%) and B-Yamagata-lineage (1592; 12%) viruses (Fig. 2A).

**Fig. 3:**
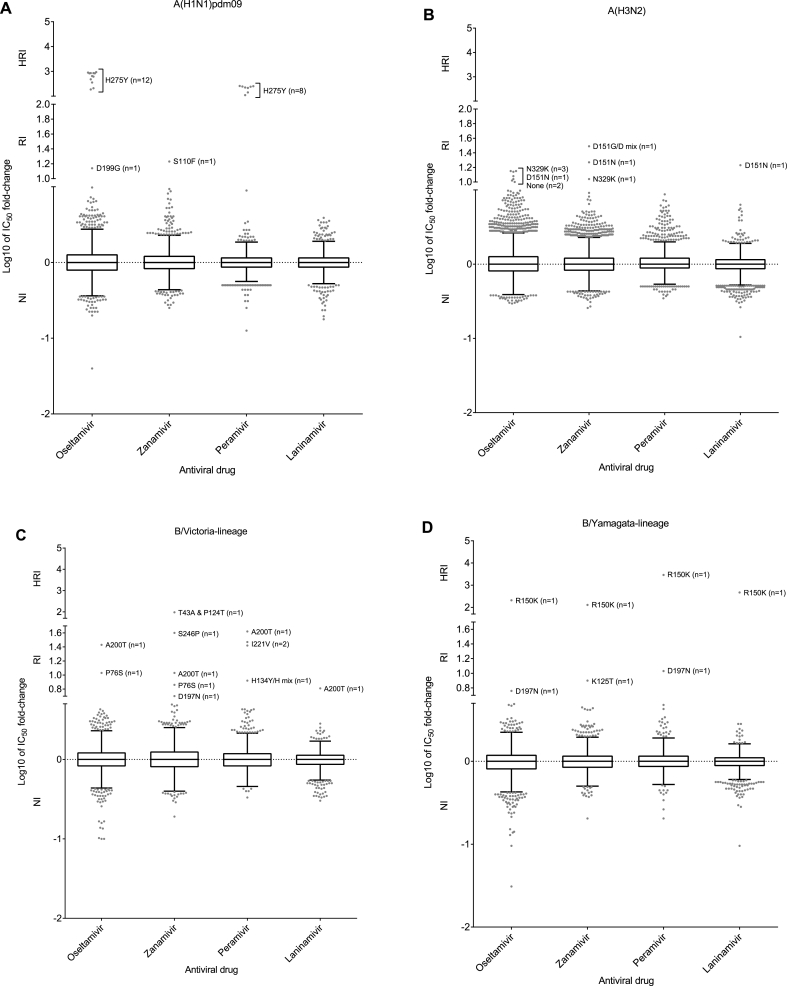
Column-scatter plots of log-transformed 50% inhibitory concentration (IC_50_) fold-change values. Data are presented by virus subtype or lineage [A) A(H1N1)pdm09; B) A(H3N2); C) B/Victoria-lineage; and D) B/Yamagata-lineage] and NAI (labelled on the X-axis: oseltamivir, zanamivir, peramivir, laninamivir). Panel C) also contains B/Yamagata-lineage haemagglutinin:B/Victoria-lineage neuraminidase reassortants. The boxes indicate the 25–75 percentile and the whiskers stretch to the lowest and highest values within 1.5 times of the interquartile region value from both the 25 and 75 percentile values respectively (Tukey's definition). The Y-axes have been split into 3 compartments according to the thresholds recommended by the World Health Organization Expert Working Group of GISRS for normal inhibition (NI) (type A viruses <10-fold; type B viruses <5-fold), reduced inhibition (RI) (type A viruses 10- to 100-fold; type B viruses 5- to 50-fold), and highly reduced inhibition (HRI) (type A viruses >100-fold; type B viruses >50-fold). NA amino acid substitutions are shown for viruses displaying RI or HRI that have been sequenced; amino acid position numbering is A subtype- and B lineage-specific. All viruses were tested for susceptibility to oseltamivir and zanamivir but only a subset were tested against peramivir and laninamivir.

A total of 32 viruses exhibiting RI/HRI by one or more NAI were detected, a rate of 0.2% (Fig. 2B; Fig. 3A–D). This is the lowest rate of RI/HRI detected since annual analysis began in 2012/13 (Fig. 2B). The number of viruses analysed is similar to that in 2014/15 and slightly lower than 2015/16 (Fig. 2A). NA sequence analysis of the 32 viruses exhibiting RI/HRI identified AAS in all but one (Table 1, Table 2, Tables S1 and S2). Clinical specimens were available for 18 of the 32 viruses, and the same AAS was confirmed in all but 2 of these (Table 1, Table 2, Tables S1 and S2).

## 3 A(H1N1)pdm09 viruses showing RI/HRI

Of the 2994 A(H1N1)pdm09 viruses tested, 14 (0.5%) exhibited RI/HRI by one or more NAI (Fig. 2B; Fig. 3A). The majority of viruses exhibiting RI/HRI contained the NA H275Y AAS (n = 12) and exhibited HRI to oseltamivir (187-941 fold-increase in IC_50_; n = 12) and peramivir (110-258 fold-increase in IC_50_; n = 8) (Table 1). These viruses were isolated from four countries; Australia (2), China (4), Japan (4) and USA (2) (Table S1). In 8 of the 12 cases, the NA H275Y AAS was confirmed in the clinical specimens, the remaining 4 cases did not have clinical specimens available for sequencing. Treatment history was available for 7 of the 12 H275Y cases; 3 had received oseltamivir treatment, while 4 had not received any NAI prior to sampling. In the 10 cases with data available, there were 4 outpatients and 6 hospitalised patients of which one patient was immuno-compromised (Table S1).

**Table 1:** Virus and patient characteristics of 21 influenza type A viruses showing reduced (RI) or highly reduced inhibition (HRI) tested by WHO CCs.^a^

Virus	n	IC_50_ fold-change compared to reference median IC_50_ values b				NA substitution c		Patient setting	Antiviral treatment	Immuno-compromised
		Oseltamivir	Zanamivir	Peramivir	Laninamivir	Virus isolate	Clinical specimen			
**A(H1N1)pdm09**; n = 2994	12	**187–941**	0.7–1.7	**110 – 258** (8)	1.5–3.4 (8)	H275Y	H275Y (8) Not available f (4)	Community (4) Hospital (6) Unknown (2)	Yes, oseltamivir (3) No (4) Unknown (5)	Yes (1) No (9) Unknown (2)
	1	9.8	**17**	n/t d	n/t	S110F	Not available	Hospital	Unknown	No
	1	**14**	8.8	2.7	2.9	D199G	D199G	Unknown	No	Unknown
**A(H3N2);** n = 6844	3	**14**	8.1–**11**	n/t	n/t	N329K	N329K (2) Not available (1)	Hospital (2) Community (1)	No (1) Unknown (2)	Unknown
	1	5.5	**31**	n/t	n/t	D151G/D mix	Not available	Hospital	Unknown	No
	1	**11**	**19**	5.3	**17**	D151N	None	Community	Yes, zanamivir	No
	1	**10.5**	3.4	n/t	n/t	S331R	S331R	Community	No	Unknown
	1	**12**	7.4	n/t	n/t	None e	None	Community	No	Unknown

a Between brackets the number of viruses for which data was reported if less than the number reported in column ‘n’.

b RI and HRI fold-change values are displayed underlined and in bold typeface.

c Amino acid position numbering is A subtype specific. The majority of samples are sequenced using next generation sequencing methodology. Precise methodology differs by WHOCC. A minority of samples are sequenced by Sanger methodology.

d n/t: not tested.

e None: no amino acid substitutions compared to the consensus sequence of viruses of the same type/subtype.

f Clinical specimen not available for sequencing.

**Table 2:** Virus and patient characteristics of 11 influenza type B viruses showing reduced (RI) or highly reduced inhibition (HRI) tested by WHO CCs.^a^

Virus	n	IC_50_ fold-change compared to reference median IC_50_ values b				NA substitution c		Patient setting	Antiviral treatment	Immuno-compromised
		Oseltamivir	Zanamivir	Peramivir	Laninamivir	Virus isolate	Clinical specimen			
**B/Victoria- lineage**; n = 2242 d	2	2.9–4.1	1.4–2.0	**26–30**	1.8–2.2	I221V (2)	Not available f (2)	Unknown (2)	Unknown (2)	Unknown (2)
	1	3.3	**96**	n/t e	n/t	T43A & P124T	Not available	Hospital	Unknown	No
	1	**11**	**7.2**	n/t	n/t	P76S	Not available	Hospital	Unknown	No
	1	2.3	1.5	**8.4**	0.8	H134Y/H mix	None g	Community	No	No
	1	3.6	**5.0**	n/t	n/t	D197N	Not available	Hospital	Unknown	No
	1	**27**	**11**	**42**	**6.4**	A200T	A200T	Unknown	Unknown	Unknown
	1	2.3	**40**	n/t	n/t	S246P	Not available	Hospital	Unknown	No
**B/Yamagata- lineage**; n = 1592	1	1.0	**7.9**	n/t	n/t	K125T	Not available	Hospital	No	Unknown
	1	**210**	**129**	**2869**	**473**	R150K	R150K	Community	No	Unknown
	1	**5.8**	4.4	**11**	2.5	D197N	D197N	Unknown	Unknown	Unknown

a Between brackets the number of viruses for which data was reported if the number reported in column ‘n’ is greater than 1.

b RI and HRI fold-change values are displayed underlined and in bold typeface.

c Amino acid position numbering is B lineage specific. The majority of samples are sequenced using next generation sequencing methodology. Precise methodology differs by WHOCC. A minority of samples are sequenced by Sanger methodology.

d Includes 47 B/Yamagata-lineage haemagglutinin (HA) – B/Victoria-lineage neuraminidase (NA) reassortants.

e n/t: not tested.

f Clinical specimen not available for testing.

g None: no amino acid substitutions compared to viruses with a normal inhibition (NI) phenotype.

One virus with NA S110F AAS, exhibiting RI by zanamivir and oseltamivir (17- and 10-fold increases in IC_50_ respectively), was isolated from an immune competent hospitalised patient whose NAI treatment history was unknown. This virus was not tested against peramivir or laninamivir and no clinical specimen was available for sequencing (Table S1). A previously described A(H1N1)pdm09 virus with S110F AAS had only 3–6 fold increases in oseltamivir and zanamivir IC_50_s compared to the mean IC_50_s (Okomo-Adhiambo et al., 2014).

Another A(H1N1)pdm09 virus, with NA D199G AAS detected in the clinical specimen and isolate, exhibited RI by oseltamivir (14-fold increase in IC_50_). The patient had not received NAI treatment but hospitalisation and immuno-competency status were unknown (Table S1). NA D199G AAS, initially associated with A(H5N1) viruses, has been described several times with the change in IC_50_ being dependent on the study and not always crossing the WHO-AVWG RI defining threshold (Gubareva et al., 2017b; Hurt et al., 2009; Pizzorno et al., 2010).

## 4 A(H3N2) viruses showing RI/HRI

Of the 6844 A(H3N2) viruses tested, 7 (0.1%) exhibited RI by one or more NAI (Fig. 2B; Fig. 3B). Three viruses with NA N329K AAS, showing borderline RI by oseltamivir and zanamivir, were detected in Europe (Table 1, Table S1). The immune status of the three patients was unknown but two were hospitalised and clinical specimens were available for two cases with the AAS being confirmed in both. NAI treatment status was unknown for two cases but was not a factor in one of the hospitalised cases.

NA S331R AAS was identified in a virus exhibiting borderline RI by oseltamivir (Table 1, Table S1). The AAS was detected in the corresponding clinical specimen but the patient, of unknown immuno-competency status and sampled from the community, had not received NAI treatment. This AAS was detected previously and the virus yielded a similarly marginal normal inhibition (NI)/RI phenotype (Takashita et al., 2015).

A virus derived from a community patient with no NAI treatment exhibited RI by oseltamivir (12-fold increase in IC_50_), but no NA AAS was identified which could explain the phenotype.

A virus from a hospitalised patient in China, with no known treatment history and no immune-compromise with a mixed AAS of D151D/G demonstrated RI (31-fold increase in IC_50_) by zanamivir but the clinical specimen was not available for sequencing. A virus from Japan showed RI by three of the four NAIs (peramivir yielded NI) and NA D151N AAS which was not seen in the corresponding clinical specimen, suggesting that this AAS was induced or selected by cell culture, or was present as a mixture below the limit of detection in the clinical specimen. This virus was isolated from a community based patient who had received zanamivir but who was not immunocompromised. While AASs have been detected at NA protein position 151 (Sheu et al., 2008; Yen et al., 2006) it is likely a cell culture artefact, particularly when using MDCK as opposed to MDCK-SIAT1 cells (Lin et al., 2010).

## 5 B/Victoria-lineage viruses showing RI/HRI

Eight of the 2242 viruses tested exhibited RI/HRI by one or more NAIs (0.4%) (Fig. 2B; Fig. 3C). The eight viruses were obtained from four countries; Mexico (2; I221V), USA (1; A200T), Japan (1; H134Y/H mixed) and China (4; P76S, D197N, T43A/P124T, S246P).

The two viruses containing NA I221V AAS both showed RI by peramivir (26-30 fold-increase in IC_50_) and NI by the other three NAIs. In both cases no clinical specimen was available and no information on the clinical setting, exposure to NAI treatment or immune status was known. The virus with NA H134H/Y mixed AAS showed RI by peramivir (8-fold increase in IC_50_); only H134 was detected in the clinical specimen collected in Japan, from a patient in the community with no links to NAI treatment. The same phenotypes caused by I221V and H134Y were reported in the previous annual summary (Gubareva et al., 2017a).

A virus with NA A200T AAS was identified in both the clinical specimen and the virus isolate, which exhibited RI by all four NAIs (6-42 fold-increases in IC_50_s), however, no patient information was known. This AAS has been identified in influenza B viruses with RI to NAIs previously, but it was thought to be cell culture-associated (Okomo-Adhiambo et al., 2014).

All four viruses from China exhibiting RI to oseltamivir and/or zanamivir were identified in isolates derived from hospitalised patients but clinical specimens were unavailable. All patients were immunocompetent but with unknown NAI treatment status. A virus with NA P76S AAS showed RI by both oseltamivir and zanamivir (11- and 7-fold increased IC_50_s respectively). Presence of D197N or S246P in two separate isolates caused RI by zanamivir (5- and 40-fold increases in IC_50_s respectively). Dual NA AAS of T43A and P124T yielded a virus with HRI by zanamivir (96-fold increase in IC_50_).

## 6 B/Yamagata-lineage viruses showing RI/HRI

Of 1592 B/Yamagata-lineage viruses tested for phenotypic susceptibility to NAIs, three showed a RI/HRI phenotype to one or more NAIs (0.2%) (Fig. 2B; Fig. 3D).

One virus with NA R150K AAS in both the isolate and the clinical specimen, collected in Taiwan from a patient in a community setting with no exposure to NAI treatment, exhibited HRI by all four NAIs (129-2869 fold-increases in IC_50_). NA D197N AAS was detected in a virus and its corresponding clinical specimen from the USA, which caused RI by oseltamivir and peramivir (6- and 11-fold increases in IC_50_s respectively). No information on the patient setting or treatment history was known. Both these AAS are in the NA active site and have been detected previously following NAI treatment (Gubareva et al., 1998; Hatakeyama et al., 2007; Ison et al., 2006).

A single isolate with NA K125T AAS, exhibited RI by zanamivir (8-fold increase in IC_50_). No clinical sample was available for testing, but the patient was hospitalised without NAI treatment. This AAS has not been identified as associating with RI to date, and it remains to be established whether K125T AAS is the cause of RI or simply present in the virus.

## 7 Frequency of RI/HRI conferring NA amino acid substitutions in sequence databases

We analysed NA sequences from viruses collected between weeks 21/2016 and 20/2017 that had been deposited in either GISAID or NCBI-IVR databases. Deduplication by strain name was performed, with preference given to sequence from original clinical material, where available. Sequences were screened for AAS known to be associated with clinical resistance, or RI/HRI in previous phenotypic studies (http://www.who.int/influenza/gisrs_laboratory/antiviral_susceptibility/avwg2014_nai_substitution_table.pdf (Gubareva et al., 2017a; Hurt et al., 2016; Meijer et al., 2014; Takashita et al., 2015).

A total of 13581 sequences were analysed, 5243 (38.6%) of which were from viruses not phenotypically tested by CCs. Screening of the additional NA sequences (452 A(H1N1)pdm09, 3420 A(H3N2), 960 B/Victoria-lineage, 411 B/Yamagata-lineage) identified a further 29 viruses with AAS associated with RI/HRI (0.55%; Table S3).

Of the 452 A(H1N1)pdm09 sequences, 5 contained H275Y AAS, associated with HRI by oseltamivir and peramivir (1.1%; Table S3). A single virus with D199G AAS was identified, as for a virus in this study which exhibited RI to oseltamivir (Table 1, Tables S1 and S3). Three viruses carried AAS at position 223, one each of I223R, I223T and I223V. Although the I223V has been shown previously to induce a <10-fold increase in IC_50_ (Hurt et al., 2009), the virus in this report also had a S247N AAS, suggesting that the dual AASs may cause RI or HRI. Four virus isolates carried NA AAS at E119K (1), D151D/E (2) and E119E/K+D151D/E. Amino acid substitutions at position 151 have been associated with cell culture-adaptation. The E119K amino acid substitution emerged following cell culture passage of a recombinant A(H1N1)pdm09 virus in the presence of laninamivir, and caused HRI by laninamivir and oseltamivir (Samson et al., 2014).

Only 4 (0.1%) of 3420 A(H3N2) NA sequences analysed had AAS associated with RI/HRI by NAIs, all with R292K, two of which were mixed populations; R292K causes HRI by all NAIs except laninamivir. Three of these viruses were from the UK, and are from patients known to have received NAI treatment. The fourth virus contained D151N AAS, in addition to R292K, both present as mixed populations. In total, 88 viruses with AAS at position 151 were identified (2.6%) and a further two with T148I (data not shown); both AAS can be cell culture-associated.

Eight (0.8%) of the additional 960 B/Victoria-lineage NA sequences carried AASs associated with RI/HRI. Two viruses had NA P139S AAS previously identified in a virus showing RI/HRI by all four NAIs but that was thought to have been selected during cell culture (Fujisaki et al., 2013). One virus had D197N AAS associated with RI by oseltamivir, zanamivir and peramivir, but a range of studies have shown variable IC_50_ values for viruses with D197N, some of which have not been greater than 5-fold increased (Gubareva et al., 1998, 2017b; Ison et al., 2006; Mishin et al., 2005). Four viruses were identified with AAS that cause RI by peramivir (K360E (1) D432G (1) and H273Y (2)) and one virus had A245T, present as a mixed population, which has been associated with RI by oseltamivir, zanamivir and peramivir (Leang et al., 2014).

Four (1%) of the additional 411 B/Yamagata-lineage NA sequences had AAS associated with RI/HRI; two with D197N, affecting all NAIs except laninamivir; one with I221V and one with I221T. I221V and I221T have been reported to cause RI by peramivir, but only I221T causes a greater than 5-fold increase in IC_50_ for oseltamivir (Gubareva et al., 2017a; Monto et al., 2006).

Finally, a comparison of the 8338 NA sequences retrieved from public databases for viruses included in phenotypic analyses identified 29 viruses of NI phenotype but which harboured AAS previously associated with RI/HRI (0.35%) (Table S4). As AAS causing RI/HRI can confer reduced viral fitness, these RI/HRI viruses could be outcompeted in cell culture by a susceptible virus, particularly if the RI variant is present in a minority proportion in the clinical sample. However, for some AASs previously detected in viruses with RI or HRI, there is limited evidence to confirm that the identified AAS is indeed responsible for the altered NAI susceptibility. Work to determine the specific impact of some of the less common AASs that have been identified is ongoing in the CCs using either reverse genetics or NA expression.

## 8 New antivirals

Numerous drugs with alternate modes of action and routes of administration are in phase III clinical trials but none have yet been licensed outside of Japan (Naesens et al., 2016).

Favipiravir is a purine nucleoside analogue, with broad spectrum antiviral activity, licenced in Japan in 2014 for use against novel influenza virus infections where existing antivirals are ineffective. Phase III trials involving uncomplicated influenza have found that favipiravir treatment reduced median time to alleviation of symptoms by 6–14 h compared to placebo (Epstein, 2017). Resistance to favipiravir has not been detected in treated patients to date, but *in vitro* passage of influenza A(H1N1)pdm09 yielded a resistant virus with an AAS in PB1, showing that resistance could emerge clinically (Lackenby, 2018).

Baloxavir is an orally administered long-acting endonuclease inhibitor, effective against influenza A and B viruses. Phase III trials in uncomplicated influenza found baloxavir, when given <48 h after onset of symptoms, lead to alleviation of symptoms 26.5 h faster than placebo and within the same time as oseltamivir (Hayden, 2018). Baloxavir-resistant variant viruses were identified in 17% of treated children during one clinical trial (Sugaya, 2018). Use of baloxavir (Xofluza ™, Shionogi) was recently approved in Japan and became available for use from the 14th March 2018 (Rana, 2018).

Pimodivir is an orally administered non-nucleotide PB2 inhibitor, acting by occupying the 7-methyl GTP cap binding site (Byrn et al., 2015). Pimodivir, effective against influenza A but not influenza B viruses due to inherent structural differences, was shown to have antiviral effects in phase IIa clinical trials in volunteers experimentally infected with influenza viruses. However, resistance was identified in 10% of cases, caused by single point mutations in the PB2 protein (Trevejo et al., 2017). Phase III trials showed that pimodivir reduced virus load alone, and to a greater degree when administered with oseltamivir; in hospitalised patients treated within 72 h of symptom onset, pimodivir plus oseltamivir eliminated detectable virus shedding 36% faster and time to return to normal activities was 61% shorter than oseltamivir alone (Hayden, 2018).

Nitazoxanide targets the mitochondria of cells infected with a virus, reducing ATP production to a level where virus replication is not possible (Rossignol and Van Baalen, 2018). In the case of influenza virus, nitazoxanide blocks the final assembly and folding of haemagglutinin at a post-translational level (Rossignol et al., 2009). Three phase IIb-III clinical trials have been completed and one is currently underway. In the first study, carried out in 624 subjects enrolled within 48 h of symptom onset, 600 mg twice daily dosage for five days was associated with a median improvement in alleviation of influenza symptoms by 22 h (p = 0.0084) and significant reduction of virus shedding (p = 0.0006) (Haffizula et al., 2014). In the second study, 1941 subjects received 600 mg nitazoxanide or 75 mg oseltamivir or 600 mg nitazoxanide plus 75 mg oseltamivir or placebo, each twice daily for 5 days. Improvements in median time to alleviation of symptoms for subjects enrolled within 36 h of symptom onset and followed up daily were 50 h for nitazoxanide (p = 0.04), 50 h for oseltamivir (p = 0.03) and 72 h for the combination (p = 0.0003) (Rossignol, 2018). The third study involving 324 patients is being analysed, and a fourth study including approximately 700 patients is underway. Nitazoxanide was well tolerated with a frequency of reported adverse events similar to placebo and resistance was not identified following serial passage of influenza viruses in increasing concentrations of the drug (Tilmanis et al., 2018).

Further phase III trials are ongoing in hospitalised and high-risk outpatients for all four drugs, with further information needed on the dosing regimen for critically ill cases, and the role of combination therapies, with NAIs and the differently acting polymerase inhibitors.

## 9 Concluding remarks

The number of viruses analysed in this global update on seasonal influenza susceptibility to NAIs () conducted by the WHO-AVWG of GISRS, was similar to 2015/16 (). The overall frequency of viruses phenotypically tested that exhibited reduced inhibition by one or more NAI was 0.2%, lower than rates observed in past analyses (2015/16: 0.8%, 2014/15: 0.5%; 2013/14: 1.9%; 2012/13: 0.6%). The proportion of A(H3N2) viruses tested in 2016/17 (50%) was similar to that in the 2014/15 (52%) and 2012/13 (45%) periods which also saw low overall proportions of viruses with RI. When the A(H1N1)pdm09 subtype dominates, as seen in 2013/14 and 2015/16 (45% and 52% of viruses tested, respectively), the overall rates of RI/HRI detection increases. In each season with widespread A(H1N1)pdm09 circulation, outbreaks of viruses harbouring NA H275Y AAS have been detected.

A ‘sequence first’ approach is being adopted by many laboratories resulting in fewer virus isolations for phenotypic characterisation. Referral of representative influenza virus-positive clinical specimens to the CCs is essential to maintain the phenotypic characterisation of viruses with NA AAS.

The frequency of viruses with RI by one of more NAIs in 2016/17 was 0.2%, the lowest incidence to date. This shows that NAIs remain appropriate for treatment and prophylaxis of seasonal influenza virus infection, but emergence of strains with RI/HRI remains a threat. The new drugs in late phase clinical trials offer the exciting prospect of a strengthened armamentarium for influenza treatment and pandemic preparedness in the near future. Development of susceptibility surveillance assays and parallel research into mechanisms of resistance to these antivirals is essential.

## Contributions

All WHO-AVWG Members and WHO Headquarters and Regional Office Staff named were involved in the development of this global update. AH/S-KL, RSD/VG, LG/LL, DW/WH, ET/TO generated and provided the NAI sensitivity data and molecular analysis. AM performed analysis of the phenotypic data from the CCs and drafted tables and figures. RTCL and SM-S performed analysis of the sequence data from GISAID and NCBI-IVR databases. AL drafted the manuscript and all authors contributed to editing the final manuscript.

## Disclaimer

The authors alone are responsible for the views expressed in this article and they do not necessarily represent the views, decisions or policies of the institutions with which they are affiliated.
