# Supplementary material for: Global update on the susceptibility of human influenza viruses to neuraminidase inhibitors and status of novel antivirals, 2016–2017
Source: Antiviral Res. 2018 Sep;157:38–46. doi: 10.1016/j.antiviral.2018.07.001 (PMC6094047; doi:10.1016/j.antiviral.2018.07.001)
Supplement: Supplementary file 1 [file mmc1.docx]

**Table S1:** Influenza A(H1N1)pdm09 and A(H3N2) virus isolates exhibiting RI or HRI by one or more NAIs (n=21).

| # | Sub-type | Strain Designation | WHO CC | Oseltamivir | Zanamivir | Peramivir | Laninamivir | Substitution in virus isolate^b^ | Substitution in original specimen^b^ | Patient setting | Antiviral treatment | Immuno- compromised | Date of collection  (y/m/d) |
| --- | --- | --- | --- | --- | --- | --- | --- | --- | --- | --- | --- | --- | --- |
|  |  |  |  | **Fold change in IC_50_^a^** | | | |  |  |  |  |  |  |
| 1 | H1N1 | A/SENDAI/85/2016 | Tokyo | **941** | 1.0 | **258** | 2.4 | H275Y | H275Y | Community | No | No | 2016-12-17 |
| 2 | H1N1 | A/SENDAI/84/2016 | Tokyo | **881** | 0.9 | **246** | 2.1 | H275Y | H275Y | Community | No | No | 2016-12-15 |
| 3 | H1N1 | A/HAWAII/25/2017 | Atlanta | **847** | 0.9 | **222** | 1.5 | H275Y | H275Y | Unknown | Unknown | Unknown | 2017-04-20 |
| 4 | H1N1 | A/SHIZUOKA-C/58/2016 | Tokyo | **842** | 1.3 | **240** | 3.4 | H275Y | H275Y | Community | No | No | 2016-08-10 |
| 5 | H1N1 | A/FUKUOKA-C/42/2016 | Tokyo | **834** | 1.2 | **215** | 3.4 | H275Y | H275Y | Community | Yes, oseltamivir | No | 2016-12-22 |
| 6 | H1N1 | A/HAWAII/20/2017 | Atlanta | **832** | 0.8 | **250** | 1.7 | H275Y | H275Y | Unknown | No | Unknown | 2017-03-26 |
| 7 | H1N1 | A/VICTORIA/2500/2016 | Melbourne | **642** | 1.3 | **111** | 2.9 | H275Y | H275Y | Hospital | Yes, oseltamivir | Yes | 2016-08-07 |
| 8 | H1N1 | A/SYDNEY/158/2016 | Melbourne | **610** | 1.0 | **141** | 2.3 | H275Y | H275Y | Hospital | Yes, oseltamivir | No | 2016-07-08 |
| 9 | H1N1 | A/Guangdong-Zhongshan/SWL1326/2017 | Beijing | **479** | 1.6 | n/t^c^ | n/t | H275Y | Not available^e^ | Hospital | Unknown | No | 2017-04-11 |
| 10 | H1N1 | A/Zhejiang-Kecheng/SWL1187/2017 | Beijing | **357** | 1.0 | n/t | n/t | H275Y | Not available | Hospital | Unknown | No | 2017-03-04 |
| 11 | H1N1 | A/Hebei-Xinhua/SWL1106/2017 | Beijing | **208** | 0.7 | n/t | n/t | H275Y | Not available | Hospital | Unknown | No | 2017-02-07 |
| 12 | H1N1 | A/Fujian-Licheng/SWL1165/2017 | Beijing | **187** | 1.7 | n/t | n/t | H275Y | Not available | Hospital | Unknown | No | 2017-02-27 |
| 13 | H1N1 | A/CALIFORNIA/193/2016 | Atlanta | **14** | 8.8 | 2.7 | 2.9 | D199G | D199G | Unknown | No | Unknown | 2016-12-24 |
| 14 | H1N1 | A/Heilongjiang-Jianhua/SWL1249/2017 | Beijing | 9.8 | **17** | n/t | n/t | S110F | Not available | Hospital | Unknown | No | 2017-03-29 |
|  |  |  |  |  |  |  |  |  |  |  |  |  |  |
| 1 | H3N2 | A/Latvia/02-062187/2017 | London | **14** | **11** | n/t | n/t | N329K | Not available | Hospital | Unknown | Unknown | 2017-02-22 |
| 2 | H3N2 | A/Poland/17C/2017 | London | **14** | 8.1 | n/t | n/t | N329K | N329K | Community | Unknown | Unknown | 2017-03-01 |
| 3 | H3N2 | A/Poland/12186/2016 | London | **14** | 9.1 | n/t | n/t | N329K | N329K | Hospital | No | Unknown | 2016-12-12 |
| 4 | H3N2 | A/Luxembourg/61798/2016 | London | **12** | 7.4 | n/t | n/t | None^d^ | None | Community | Unknown | Unknown | 2016-12-02 |
| 5 | H3N2 | A/CHIBA-C/96/2016 | Tokyo | **11** | **19** | 5.3 | **17** | D151N | None | Community | Yes, zanamivir | No | 2016-09-08 |
| 6 | H3N2 | A/Ireland/81104/2016 | London | **10.5** | 3.4 | n/t | n/t | S331R | S331R | Community | No | Unknown | 2016-12-15 |
| 7 | H3N2 | A/Guangdong-Zhenjiang/1326/2017 | Beijing | 5.5 | **31** | n/t | n/t | D151G/D mix | Not available | Hospital | Unknown | No | 2017-04-18 |

^a^ RI and HRI fold-change values are displayed underlined and in bold typeface.

^b^ Amino acid position numbering is A subtype specific. The majority of samples are sequenced using next generation sequencing methodology. Precise methodology differs by WHOCC. A minority of samples are sequenced by Sanger methodology.

^c^ n/t: not tested.

^d^ None: no amino acid substitutions compared to viruses with NI phenotype.

^e^ Clinical specimen not available for sequencing.
