# Supplementary material for: Global update on the susceptibility of human influenza viruses to neuraminidase inhibitors and status of novel antivirals, 2016–2017
Source: Antiviral Res. 2018 Sep;157:38–46. doi: 10.1016/j.antiviral.2018.07.001 (PMC6094047; doi:10.1016/j.antiviral.2018.07.001)
Supplement: Supplementary file 2 [file mmc2.docx]

**Table S2:** Influenza B/Victoria/2/87- lineage and Influenza B/Yamagata/16/88- lineage viruses exhibiting RI or HRI by one or more NAIs (n=11).

| # | Lineage | Strain Designation | WHO CC | Oseltamivir | Zanamivir | Peramivir | Laninamivir | Substitution in virus isolate^b^ | Substitution in original specimen^b^ | Patient setting | | Antiviral treatment | | Immuno- compromised | | Date of collection  (y/m/d) | |
| --- | --- | --- | --- | --- | --- | --- | --- | --- | --- | --- | --- | --- | --- | --- | --- | --- | --- |
|  |  |  |  | **Fold change in IC_50_^a^** | | | |  |  |  |  |  |  |  |  |  |  |
| 1 | B Victoria | B/FLORIDA/103/2016 | Atlanta | **27** | **11** | **42** | **6.4** | A200T | A200T | Unknown | | Unknown | | Unknown | | 2016-12-19 | |
| 2 | B Victoria | B/Jiangsu-Gaoyou/1396/2017 | Beijing | **11** | **7.2** | n/t^c^ | n/t | P76S | Not available^d^ | Hospital | | Unknown | | No | | 2017-04-19 | |
| 3 | B Victoria | B/MEXICO/4260/2016 | Atlanta | 4.1 | 1.4 | **30** | 1.8 | I221V | Not available | Unknown | | Unknown | | Unknown | | 2016-08-18 | |
| 4 | B Victoria | B/Fujian-Yanping/1581/2016 | Beijing | 3.6 | **5.0** | n/t | n/t | D197N | Not available | Hospital | | Unknown | | No | | 2016-07-05 | |
| 5 | B Victoria | B/Sichuan-Dongxin/1297/2017 | Beijing | 3.3 | **96** | n/t | n/t | T43A,P124T | Not available | Hospital | | Unknown | | No | | 2017-03-20 | |
| 6 | B Victoria | B/MEXICO/209/2017 | Atlanta | 2.9 | 2.0 | **26** | 2.2 | I221V | Not available | Unknown | | Unknown | | Unknown | | 2017-01-16 | |
| 7 | B Victoria | B/SAGAMIHARA/30/2017 | Tokyo | 2.3 | 1.5 | **8.4** | 0.8 | H134Y/H mix | None | Community | | No | | No | | 2017-03-27 | |
| 8 | B Victoria | B/Yunnan-Maoshi/1821/2016 | Beijing | 2.3 | **40** | n/t | n/t | S246P | Not available | Hospital | | Unknown | | No | | 2016-10-17 | |
|  |  |  |  |  |  |  |  |  |  |  |  | |  | |  | |  |
| 1 | B Yamagata | B/Taiwan/56/2017 | Tokyo | **210** | **129** | **2869** | **473** | R150K | R150K | Community | No | | Unknown | | 2017-05-19 | |  |
| 2 | B Yamagata | B/WYOMING/07/2017 | Atlanta | **5.8** | 4.4 | **11** | 2.5 | D197N | D197N | Unknown | Unknown | | Unknown | | 2017-03-27 | |  |
| 3 | B Yamagata | B/Latvia/04-014585/2017 | London | 1.0 | **7.9** | n/t | n/t | K125T | Not available | Hospital | No | | Unknown | | 2017-04-05 | |  |

^a^ RI and HRI fold-change values are displayed underlined and in bold typeface.

^b^ Amino acid position numbering is B lineage specific. The majority of samples are sequenced using next generation sequencing methodology. Precise methodology differs by WHOCC. A minority of samples are sequenced by Sanger methodology.

^c^ n/t: not tested.

^d^ Clinical specimen not available for sequencing.
