# Supplementary material for: Global update on the susceptibility of human influenza viruses to neuraminidase inhibitors and status of novel antivirals, 2016–2017
Source: Antiviral Res. 2018 Sep;157:38–46. doi: 10.1016/j.antiviral.2018.07.001 (PMC6094047; doi:10.1016/j.antiviral.2018.07.001)
Supplement: Supplementary file 3 [file mmc3.docx]

**Table S3:** Influenza types A and B viruses (n=29) with unavailable NAI susceptibility data carrying NA substitutions associated with RI/HRI

| # | Designation | Submitting laboratory | A subtype  B lineage | Passage details/ history^a^ | NA  AAS ^b^ | NA GISAID  Acc. No. | Country of specimen collection |
| --- | --- | --- | --- | --- | --- | --- | --- |
| 1 | A/Guizhou-Qingzhen/SWL1353/2017 | WHO Chinese National Influenza Center | A(H1N1)pdm09 | E2+E1 | E119K | EPI1029921 | China |
| 2 | A/Denmark/53/2016 | Crick Worldwide Influenza Centre | A(H1N1)pdm09 | SIAT3/MDCK1 | E119E/K; D151D/E | EPI917122 | Denmark |
| 3 | A/Guizhou-Qingzhen/1331/2017 | Centers for Disease Control and Prevention | A(H1N1)pdm09 | C3 | D151D/E | EPI1058874 | China |
| 4 | A/Odessa/166/2017 | Centers for Disease Control and Prevention | A(H1N1)pdm09 | CXSX | D151D/E | EPI1017534 | Ukraine |
| 5 | A/Sichuan-Anyue/SWL77/2017 | WHO Chinese National Influenza Center | A(H1N1)pdm09 | C1+C1 | D199G | EPI1062840 | China |
| 6 | A/California/NHRC_NMCSD0076/2016 | Other Database Import | A(H1N1)pdm09 | MDCK-SIAT1 | I223R | EPI1030711 | United States |
| 7 | A/Hong Kong/2203/2017 | Crick Worldwide Influenza Centre | A(H1N1)pdm09 | cs | I223T | EPI1019932 | Hong Kong (SAR) |
| 8 | A/Guangdong-Dongwanbendi/SWL1314/2017 | WHO Chinese National Influenza Center | A(H1N1)pdm09 | E3+E1 | 1223V; S247N | EPI1062822 | China |
| 9 | A/Fujian-Tongan/SWL1341/2017 | WHO Chinese National Influenza Center | A(H1N1)pdm09 | C4+C1 | H275Y | EPI1062843 | China |
| 10 | A/ London/16U426028-92_S44_L001/2016 | University College London | A(H1N1)pdm09 | Not given | H275Y | EPI1041349 | United Kingdom |
| 11 | A/Paris/1227/2017 | Crick Worldwide Influenza Centre | A(H1N1)pdm09 | MDCK1/MDCK1 | H275Y | EPI1041822 | France |
| 12 | A/Quebec/RV1925/2017 | Public Health Agency of Canada (PHAC) | A(H1N1)pdm09 | P1 | H275Y | EPI1006448 | Canada |
| 13 | A/Singapore/KK0254/2017 | Ministry of Health, Singapore | A(H1N1)pdm09 | cs | H275Y | EPI1023281 | Singapore |
| 1 | A/Banska Bystrica/363/2017 | Crick Worldwide Influenza Centre | A(H3N2) | MDCKx/SIAT1 | R292R/K; D151D/N | EPI1046384 | Slovakia |
| 2 | A/England/65020628/2016 | Microbiology Services Colindale, Public Health England | A(H3N2) | original | R292K | EPI1030364 | United Kingdom |
| 3 | A/England/65280702/2016 | Microbiology Services Colindale, Public Health England | A(H3N2) | original | R292K | EPI1030384 | United Kingdom |
| 4 | A/England/70660207/2017 | Microbiology Services Colindale, Public Health England | A(H3N2) | original | R292R/K | EPI1024709 | United Kingdom |
| 1 | B/Astana/ZVL229/2016 | National Centre of Expertise in Astana | B Victoria | c1 | P139S | EPI1005997 | Kazakhstan |
| 2 | B/Uralsk/ZVL399/2016 | National Centre of Expertise in Astana | B Victoria | c1 | P139S | EPI1006007 | Kazakhstan |
| 3 | B/Florida/63/2017 | Centers for Disease Control and Prevention | B Victoria | Original | D197N | EPI1021044 | United States |
| 4 | B/Hong Kong/219/2017 | Crick Worldwide Influenza Centre | B Victoria | cs | A245A/T | EPI1022733 | Hong Kong (SAR) |
| 5 | B/Malaysia/1191/2016 | WHO Collaborating Centre for Reference and Research on Influenza | B Victoria | X,MDCK1 | H273Y; D149D/N | EPI869040 | Malaysia |
| 6 | B/Moscow/107/2017 | Crick Worldwide Influenza Centre | B Victoria | MDCK2 | H273Y | EPI1040352 | Russian Federation |
| 7 | B/Arizona/49/2017 | Centers for Disease Control and Prevention | B Victoria | Original | K360E | EPI1051346 | United States |
| 8 | B/Brisbane/240/2015 | WHO Collaborating Centre for Reference and Research on Influenza | B Victoria | MDCK2 | D432G | EPI869013 | Australia |
| 1 | B/England/71280549/2017 | Microbiology Services Colindale, Public Health England | B Yamagata | original | D197N | EPI1030586 | United Kingdom |
| 2 | B/Ontario/RV1275/2017 | Public Health Agency of Canada (PHAC) | B Yamagata | P1 | D197N | EPI1004665 | Canada |
| 3 | B/Quebec/RV0463/2017 | Public Health Agency of Canada (PHAC) | B Yamagata | P1 | I221T | EPI1005692 | Canada |
| 4 | B/Brazil/236/2017 | Centers for Disease Control and Prevention | B Yamagata | Original | I221V | EPI1052695 | Brazil |

^a^ Passage as shown in the sequence databases.

^b^ NA amino acid numbering is subtype/lineage-specific. The majority of samples are sequenced using next generation sequencing methodology. Precise methodology differs by WHOCC. A minority of samples are sequenced by Sanger methodology. NA amino acid substitutions (AAS) associated with RI/HRI, as listed in the summary table provided by the AVWG on the WHO website (<http://www.who.int/influenza/gisrs_laboratory/antiviral_susceptibility/avwg2014_nai_substitution_table.pdf> ) are shown.
