# Supplementary material for: Global update on the susceptibility of human influenza viruses to neuraminidase inhibitors and status of novel antivirals, 2016–2017
Source: Antiviral Res. 2018 Sep;157:38–46. doi: 10.1016/j.antiviral.2018.07.001 (PMC6094047; doi:10.1016/j.antiviral.2018.07.001)
Supplement: Supplementary file 4 [file mmc4.docx]

**Table S4:** Influenza types A and B viruses (n=29) showing NI but carrying NA substitutions associated with RI/HRI

| # | Designation | Submitting laboratory | A subtype  B lineage | Passage details/ history^a^ | NA  AAS ^b^ | NA GISAID  Acc. No. | Country of specimen collection |
| --- | --- | --- | --- | --- | --- | --- | --- |
| 1 | A/Brisbane/130/2016 | WHO Collaborating Centre for Reference and Research on Influenza | A(H1N1)pdm09 | MDCK2 | E119E/K | EPI829210 | Australia |
| 2 | A/KOCHI/2/2017 | National Institute of Infectious Diseases (NIID) | A(H1N1)pdm09 | MDCK 2 +1 | E119E/K | EPI1002857 | Japan |
| 3 | A/Laos/F4608/2016 | National Institute of Infectious Diseases (NIID) | A(H1N1)pdm09 | MDCK 1 +1 | E119E/K | EPI1027162 | Lao, People's Democratic Republic |
| 4 | A/Shanghai-Fengxian/SWL1418/2017 | WHO Chinese National Influenza Center | A(H1N1)pdm09 | E1+E1 | E119E/K | EPI1029933 | China |
| 5 | A/SHIMANE/94/2017 | National Institute of Infectious Diseases (NIID) | A(H1N1)pdm09 | MDCK 1 +1 | E119E/K; D151D/N/G | EPI1044570 | Japan |
| 6 | A/South Auckland/33/2016 | WHO Collaborating Centre for Reference and Research on Influenza | A(H1N1)pdm09 | MDCKX, MDCK1 | E119E/K; D151D/N | EPI829261 | New Zealand |
| 7 | A/MIYAGI/39/2016 | National Institute of Infectious Diseases (NIID) | A(H1N1)pdm09 | MDCK 2 +1 | Q136Q/K | EPI970759 | Japan |
| 8 | A/Shanghai-Xuhui/SWL1246/2017 | WHO Chinese National Influenza Center | A(H1N1)pdm09 | C1+C1 | I223T | EPI1030158 | China |
| 9 | A/Liaoning-Zhenxin/SWL1114/2017 | WHO Chinese National Influenza Center | A(H1N1)pdm09 | C2+C1 | S247G | EPI1030305 | China |
| 10 | A/Uruguay/330/2016 | Centers for Disease Control and Prevention | A(H1N1)pdm09 | C2 | H275H/Y | EPI840890 | Uruguay |
| 1 | A/Newcastle/205/2016 | WHO Collaborating Centre for Reference and Research on Influenza | A(H3N2) | SIAT1 | Q391K | EPI868917 | Australia |
| 1 | B/Myanmar/16M010/2016 | National Institute of Infectious Diseases (NIID) | B Victoria | MDCK 2 +1 | E105E/K | EPI977402 | Myanmar |
| 2 | B/SAGA/187/2016 | National Institute of Infectious Diseases (NIID) | B Victoria | MDCK 3 +1 | E105E/K; G145G/R | EPI909517 | Japan |
| 3 | B/KOCHI/102/2017 | National Institute of Infectious Diseases (NIID) | B Victoria | MDCK 1 +1 | P139P/S | EPI1004827 | Japan |
| 4 | B/KANAGAWA/AC1637/2017 | National Institute of Infectious Diseases (NIID) | B Victoria | MDCK 0 +1 | D432D/G | EPI975847 | Japan |
| 5 | B/Hubei-Shashi/1244/2017 | WHO Chinese National Influenza Center | B Victoria | C1+C1 | D197E | EPI1029939 | China |
| 6 | B/Arizona/06/2017 | Other Database Import | B Victoria | Original | K360E | EPI968964 | United States |
| 7 | B/EHIME/3/2017 | National Institute of Infectious Diseases (NIID) | B Victoria | MDCK 2 +1 | K360E | EPI1033133 | Japan |
| 8 | B/KITAKYUSYU/5/2017 | National Institute of Infectious Diseases (NIID) | B Victoria | MDCK 1 +1 | K360E | EPI1033153 | Japan |
| 9 | B/SAITAMA/82/2017 | National Institute of Infectious Diseases (NIID) | B Victoria | Original | K360E | EPI1055149 | Japan |
| 10 | B/Singapore/GP2355/2016 | Ministry of Health, Singapore | B Victoria | Clinical specimen | K360E | EPI864711 | Singapore |
| 1 | B/TOKYO/16590/2017 | National Institute of Infectious Diseases (NIID) | B Yamagata | MDCK 1 +1 | E117E/G | EPI943365 | Japan |
| 2 | B/KAGAWA/160/2017 | National Institute of Infectious Diseases (NIID) | B Yamagata | MDCK 1 +1 | H134H/Y | EPI1027373 | Japan |
| 3 | B/New Jersey/25/2017 | Other Database Import | B Yamagata | Original | K152N | EPI1044180 | United States |
| 4 | B/Chongqing-Yuzhong/1639/2017 | WHO Chinese National Influenza Center | B Yamagata | C1+C1 | R186K;I262T | EPI1030107 | China |
| 5 | B/Hubei-Echeng/11839/2017 | WHO Chinese National Influenza Center | B Yamagata | C2+C1 | R186K;I262T | EPI1030116 | China |
| 6 | B/Hunan-Yuhua/1692/2017 | WHO Chinese National Influenza Center | B Yamagata | C1+C1 | R186K;I262T | EPI1030134 | China |
| 7 | B/Yokohama/112/2016 | Centers for Disease Control and Prevention | B Yamagata | C2C2 | R186K;I262T | EPI918026 | Japan |
| 8 | B/YOKOHAMA/113/2016 | National Institute of Infectious Diseases (NIID) | B Yamagata | MDCK 2 +1 | R186K;I262T | EPI881881 | Japan |

^a^ Passage as shown in the sequence databases.

^b^ NA amino acid numbering is subtype/lineage-specific. The majority of samples are sequenced using next generation sequencing methodology. Precise methodology differs by WHOCC. A minority of samples are sequenced by Sanger methodology. NA amino acid substitutions (AAS) associated with RI/HRI, as listed in the summary table provided by the AVWG on the WHO website (<http://www.who.int/influenza/gisrs_laboratory/antiviral_susceptibility/avwg2014_nai_substitution_table.pdf> ), are shown.
